# Effects of vasodilators on beat-to-beat and every fifteen minutes blood pressure variability induced by noradrenaline infusion in rats

**DOI:** 10.1038/s41440-024-01595-w

**Published:** 2024-02-09

**Authors:** Danfeng Jiang, Minami Matsuzaki, Takanori Ida, Kazuo Kitamura, Johji Kato

**Affiliations:** Frontier Science Research Center, University of Miyazaki Faculty of Medicine, Miyazaki, 889-1692 Japan

**Keywords:** Blood pressure variability, Noradrenaline, Azelnidipine, Hydralazine, Baroreceptor reflex sensitivity

## Abstract

Increased blood pressure variability (BPV) was shown to be associated with cardiovascular morbidities and/or mortalities. There are various types of BPV depending on time intervals of BP measurements, ranging from beat-to-beat to visit-to-visit or year-to-year. We previously found that continuous infusion of noradrenaline (NA) for 14 days increased short-term BPV every 15 min in rats. The aims of this study were to examine (1) whether NA infusion increases very short-term beat-to-beat BPV, (2) the effects of azelnidipine and hydralazine on NA-induced BPV, and (3) whether baroreceptor reflex sensitivity (BRS) is affected by NA or NA plus those vasodilators. Nine-week-old Wistar rats infused subcutaneously with 30 μg/h NA were orally treated with or without 9.7 mg/day azelnidipine or 5.9 mg/day hydralazine over 14 days. BP levels were continuously monitored via abdominal aortic catheter with a telemetry system in an unrestrained condition. Standard deviations (SDs) were used to evaluate beat-to-beat BPV and BPV every 15 min which was obtained by averaging BP levels for 10-s segment at each time point. BRS was determined by a sequence analysis. Continuous NA infusion over 14 days increased average BP, beat-to-beat BPV, and BPV every 15 min, lowering BRS. Comparing the two vasodilators, hydralazine reduced BP elevation by NA; meanwhile, azelnidipine alleviated BPV augmentation, preserving BRS, despite a smaller BP reduction. Thus, NA infusion increased both very short- and short-term BPV concomitantly with impaired BRS, while azelnidipine had an inhibitory effect, possibly independent of BP-lowering, on those types of BPV and impairment of BRS.

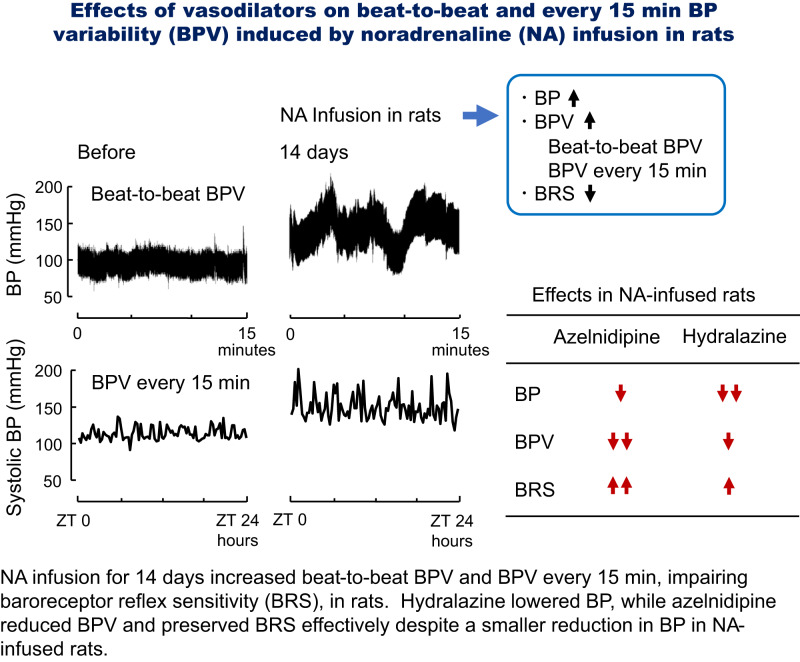

## Introduction

Hypertension is a world-wide health concern because of its close association with cardiovascular and renal diseases. In addition to blood pressure (BP) elevation, clinical studies revealed fluctuation of BP levels within a certain period of time, so called blood pressure variability (BPV), to be an independent cardiovascular risk [1–6]. There are various types of BPV depending on length of time period, during which variability is evaluated, from very short-term beat-to-beat BPV to long-term visit-to-visit or year-to-year BPV [3, 6]. Irrespective of time period, a consistent finding is that the larger the variability, the higher the cardiovascular mobility or mortality [1–6]. On the other hand, a large number of clinical studies have been carried out to specify drugs that effectively alleviate augmented BPV [7–10]. Either monotherapy with calcium channel blocker (CCB) or a combination of CCB and renin-angiotensin system inhibitor was reported to be effective in reducing BPV as compared with other classes or combinations of anti-hypertensive agents [7–10].

Various animal models of hypertension or cardiovascular diseases have been used to explore the pathophysiology and/or therapeutic strategies for those diseases, while there have been a limited number of animal models available for augmented BPV [11–14]. Recently, we reported that BPV is augmented in rats following continuous 14-day infusion of either angiotensin II (Ang II) or noradrenaline (NA) [13–15]. In those studies, our efforts were made to develop animal models for augmented BPV in 24-h ambulatory BP monitoring of human patients. Therefore, we evaluated BP levels at intervals of 15 min, which were obtained by averaging BP monitored for 10-s segment at each time point [13–15]; meanwhile, very short-term beat-to-beat BPV remains to be determined in those rat models. The present study was carried out to examine (1) whether continuous NA infusion increases very short-term beat-to-beat BPV as it does for BPV every 15 min, (2) the effects of the vasodilators azelnidipine and hydralazine on NA-induced BPV, and (3) whether baroreceptor reflex sensitivity (BRS) is affected by NA infusion with or without treatment with those vasodilators.

## Methods

### Animals and chemicals

Animals used in this study were 8-week-old male Wistar rats weighing 318 ± 30 g (mean ± SD), which had been purchased from Jackson Laboratory Japan, Yokohama, Japan. The rats were maintained under a 12-h light and 12-h dark cycle in a specific pathogen-free condition with standard chow and water given ad libitum during the entire experiment period, including accommodation to new environment, in the Divisions of Bioresources, Frontier Science Research Center, University of Miyazaki. L-noradrenaline bitartrate monohydrate, azelnidipine, and hydralazine were obtained from Tokyo Chemical Industry Co., Ltd. (Tokyo, Japan). The present study was performed in accordance with the Animal Welfare Act and with approval of the University of Miyazaki Institutional Animal Care and Use Committee (No. 2017-509 and No. 2023-502).

### BP monitoring and experiment protocol

BP levels were monitored using a telemetry system (Data Sciences International, St. Paul, MN, USA) with the HD-S10 transmitter implanted in the abdominal cavity, as previously described [13–15]. In brief, under anesthesia by intraperitoneal injection of 2.0 mg/kg butorphanol tartrate, 1.6 mg/kg midazolam, and 0.12 mg/kg medetomidine hydrochloride, the abdominal cavity was opened by a middle incision and the aorta was isolated from the retroperitoneal tissue. The catheter inserted into the aorta was secured with tissue adhesive and the transmitter was then placed in the intraperitoneal space by suturing to the muscular layer of the abdominal wall. Before closing the wall, abdominal cavity was filled with 4 to 5 ml of prewarmed sterile saline to avoid post-operative hypovolemia and hypothermia.

After the 7-day period of recovery from the implantation operation, rats were divided into four groups infused with the vehicle or NA solution with or without oral administration of azelnidipine or hydralazine over 14 days. NA dissolved in distilled water containing 0.005% ascorbic acid and 5 mM glutathione was subcutaneously infused into rats at a rate of 30 μg/h, over 14 days via an osmotic mini-pump (Alzet Model 2002; Durect, Cupertino, CA, USA). We intended to administer azelnidipine at 30 mg/kg/day and hydralazine at 20 mg/kg/day in rat chow and drinking water, respectively. According to our pilot studies, averages of daily chow and water consumption were found to be about 20 g/day and 50 ml/day, respectively, in Wistar rats that we use in our laboratory. Based on these results, azelnidipine was given at 0.45 mg per gram of rat chow and hydralazine was administered at 0.133 mg per milliliter of water. The numbers of rats examined were as follows: control, 8; NA, 9; NA plus azelnidipine, 8; NA plus hydralazine, 7.

The 24-h BP recordings were carried out with rats in a conscious, unrestrained condition, before and after the infusion. Ponemah software (Data Sciences International) was used to calculate averages and standard deviations (SDs) of all the values of systolic and diastolic BP (SBP and DBP) continuously monitored during the 12-h light and 12-h dark cycles; very short-term beat-to-beat BPV was evaluated with those SD data. In the meantime, BPV every 15 min was determined as described previously [13–15]. In brief, averages of SBP and DBP during 10-s segment at each time point were obtained, and then, variability was evaluated with SDs of these BP data of the light and dark cycles (48 points for each). After the BP recording at day 14, the animals were euthanized by drawing the whole blood from the inferior vena cava under anesthesia described above.

### Calculation of baroreceptor reflex sensitivity (BRS)

To estimate BRS, data obtained from continuous BP monitoring were analyzed by a sequence technique with the software HemoLab before and after 14 days of the experiment period, as reported previously [16–18] This method identified spontaneous sequences of four or more heart beats, where BP levels and pulse intervals changing in the same direction were linearly correlated at an *r* value greater than 0.8, and the mean of slopes of regression lines was used as an index of BRS. Averages of BRS values calculated from the continuous BP monitoring data during the time periods of ZT 2 to 6 (10:00–14:00) and ZT 14 to 18 (22:00–2:00) were obtained in the present study.

### Statistical analysis

All data were analyzed statistically with IBM SPSS software version 29.0 (IBM, Armonk, NY, USA). Multiple comparisons were made with one-way analysis of variance and the Tukey-Kramer method. Simple regression analysis was used to examine the relationship between two variables. All data are expressed as the mean ± standard error of the mean (SEM), otherwise indicated, and *P* < 0.05 was considered to be significant.

## Results

**Fig. 1:**
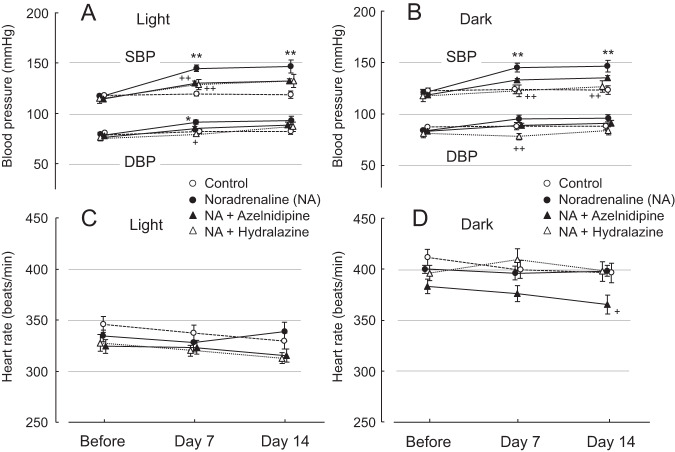
Averages of systolic and diastolic blood pressure (SBP and DBP) and heat rate values during 12-h time period of the light (**A**, **C**) and dark (**B**, **D**) cycles before and 7 and 14 days of vehicle or NA infusion with or without oral administration of azelnidipine or hydralazine. Mean ± SEM; *P < 0.05, **P < 0.01, vs. control group; ^+^*P* < 0.05, ^++^*P* < 0.01, vs. NA group without vasodilator treatment. Average values during the light and dark cycles are also shown numerically in Supplementary Tables 1 and 2, respectively

Figure 1 shows averages of SBP and DBP and those of heart rate in rats infused continuously with NA over 14 days, that were treated with or without azelnidipine or hydralazine. NA infusion increased SBP and DBP at the light and dark cycles, while NA had no significant effects on heart rate, as compared with the control. Treatments with azelnidipine and hydralazine lowered SBP and DBP, while these effects were partially insignificant. When comparing the BP-lowering effects of two vasodilators, the latter was more effective than the former particularly in the dark cycle (Fig. 1B). The results of Fig. 1 are shown as numerical data in Supplementary Tables 1 and 2. Neither azelnidipine nor hydralazine had a significant effect on heart rate in NA-infused rats, except for that of the azelnidipine group at the dark cycle of day 14 (Fig. 1D). Averages of daily chow and water consumption in rats treated with azelnidipine and hydralazine were 21.6 ± 0.6 g/day and 44.1 ± 1.2 ml/day, respectively. Therefore, doses of azelnidipine and hydralazine given to the animals were calculated to be 9.7 ± 0.3 mg/day and 5.9 ± 0.2 mg/day, respectively, during the experimental period. In addition, we collected data on locomotive activities from individual animals via this telemetry system. The activities of four groups at the dark cycle were higher than those at the light, but no differences were noted between the study groups at both cycles.

**Fig. 2:**
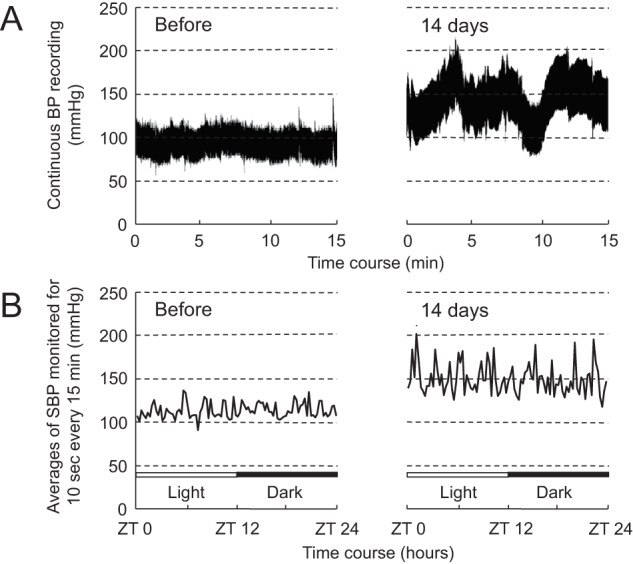
Representative graphs of very short-term (**A**) and short-term (**B**) blood pressure variabilities (BPVs) before and after 14 days of NA infusion. The graphs (**A**) and (**B**) show continuous BP recordings for a 15 min time period (**A**) and BP levels every 15 min in the 12-h light and 12-h dark cycles (**B**), respectively. The data of very short-term BPV before and after 14 days of NA infusion (**A**) were taken from the light cycles of ZT 10:21–10:36 (18:21–13:36) and ZT 10:12–10:27 (18:12–18:27), respectively

Figure 2A, B are representative graphs of BP recordings for very short-term beat-to-beat BPV over 15 min and short-term BPV at intervals of 15 min over 24 h, respectively. As shown in Fig. 2A, NA infusion for 14 days increased not only average BP level but also beat-to-beat fluctuation of BP. Similarly, SBP levels recorded every 15 min were higher and more widely fluctuated following NA infusion for 14 days, compared with those before the infusion (Fig. 2B).

**Fig. 3:**
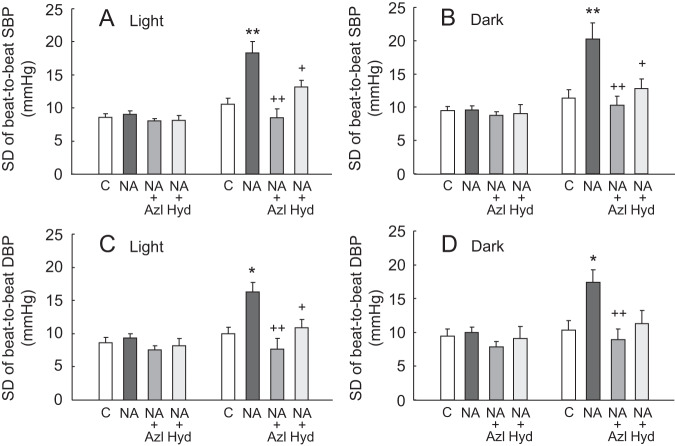
Standard deviations (SDs) of beat-to-beat systolic and diastolic blood pressure (SBP and DBP) values continuously recorded during 12-h time period of the light (**A**, **C**) and dark (**B**, **D**) cycles before and after 14 days of vehicle or NA infusion with or without oral administration of azelnidipine (Azl) or hydralazine (Hyd). Mean ± SEM; *P < 0.05, **P < 0.01, vs. control group; ^+^*P* < 0.05, ^++^*P* < 0.01, vs. NA group without vasodilator treatment. SDs were calculated from all the values of SBP and DBP recorded continuously during 12-h time period of the light and dark cycles

**Fig. 4:**
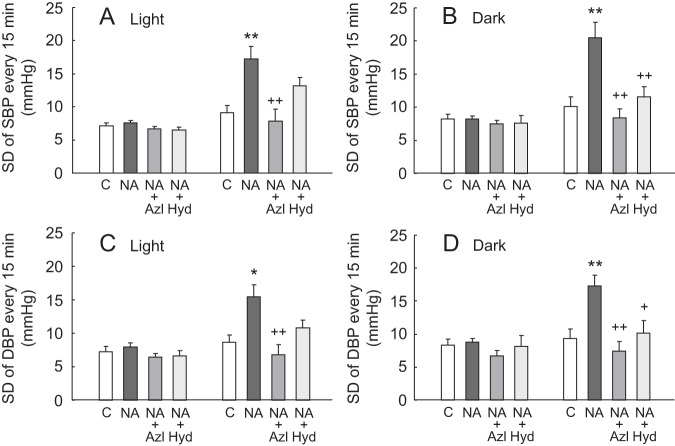
Standard deviations (SDs) of systolic and diastolic blood pressure (SBP and DBP) values recorded every 15 min during 12-h time period of the light (**A**, **C**) and dark (**B**, **D**) cycles before and after 14 days of vehicle or NA infusion with or without oral administration of azelnidipine (Azl) or hydralazine (Hyd). Mean ± SEM; *P < 0.05, **P < 0.01, vs. control group; ^+^*P* < 0.05, ^++^*P* < 0.01, vs. NA group without vasodilator treatment. SDs were calculated from SBP and DBP values obtained by averaging BP data recorded for 10-second segments at time points every 15 min (48 points per 12 h)

Figure 3 shows SDs of beat-to-beat SBP and DBP levels monitored during the 12-h light and 12-h dark cycles. Beat-to-beat variabilities of SBP and DBP were augmented by NA infusion for 14 days, but these augmentations in BPV were lowered almost to control levels following treatment with azelnidipine. Hydralazine also alleviated NA-induced beat-to-beat variabilities, while the effect was smaller than azelnidipine and partially insignificant. BPVs at 15 min intervals are summarized in Fig. 4, where results similar to those of beat-to-beat BPV were obtained.

**Fig. 5:**
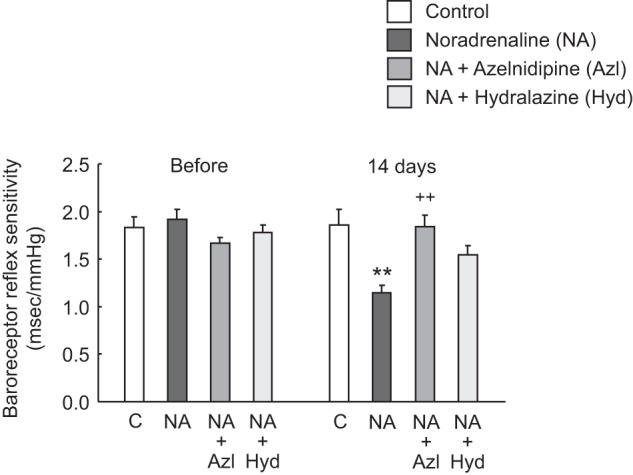
Baroreceptor reflex sensitivities (BRSs) before and after 14 days of vehicle or NA infusion with or without oral administration of azelnidipine (Azl) or hydralazine (Hyd). Mean ± SEM; **P < 0.01, vs. control group; ^++^*P* < 0.01, vs. NA group without vasodilator treatment

Figure 5 shows BRS values of the four groups before and after NA infusion for 14 days with or without treatment with azelnidipine or hydralazine. NA infusion significantly lowered BRS, while this reduction was mostly inhibited by azelnidipine. Hydralazine also inhibited NA-induced reduction in BRS, but the effect was statistically insignificant. Then, we looked at whether BRS values were correlated with BPV using a simple regression analysis. When data from the four study groups were analyzed together, BRS values at day 14 showed a significant inverse correlation with both beat-to-beat BPV and BPV every 15 min of SBP and DBP at the light and dark cycles, displaying correlation coefficients (*r*) of -0.396 to -0.535 and *P* values of 0.025 to 0.002.

## Discussion

Previously, we found that continuous NA infusion increased short-term BPV in an attempt to develop rat models of increased BPV seen in 24-h ambulatory BP monitoring of human patients [13–15]. In that experiment, we evaluated variability with BP levels obtained every 15 min by averaging BP monitored for 10-s segment at each time point [13–15]. New findings in the present study are as follows: first, NA infusion over 14 days increased very short-term beat-to-beat BPV as it did for BPV every 15 min. Second, when comparing azelnidipine and hydralazine, the former effectively inhibited augmentation of beat-to-beat BPV and BPV every 15 min despite a smaller reduction in average BP levels. Third, NA infusion resulted in impaired BRS, while this impairment was significantly alleviated by treatment with azelnidipine. Thus, we collected a wide range of data that are essential for discussing the mechanisms for augmented BPV and/or actions of vasodilators by a single set of animal experiments in this study.

Because augmented BPV was found to be closely associated with elevation of BP in patients with hypertension [3], we previously looked at short-term BPVs every 15 min in rat models of hypertension: spontaneously hypertensive rats (SHR) and those continuously infused with Ang II or NA [13, 14]. BPV every 15 min in SHR was found to be higher than that in the control WKY rats, but when evaluated by coefficients of variation, this difference in BPV became unclear. Meanwhile, BPVs in rats infused with Ang II or NA were clearly augmented than those seen in SHR [13, 14]. These findings are consistent with the notion that not only BP elevation but also humoral factors, such as Ang II or NA, are involved in augmented BPV [3].

It was reported that short-term beat-to-beat BPV is closely associated with BP regulation by baroreflex [19], and in accordance with this, a significant correlation was noted between beat-to-beat BPV and BRS in the present study. In addition, BPV every 15 min was found to be associated with BRS, suggesting that not only beat-to-beat but also BPV every 15 min is affected by baroreflex regulation of BP. An important point of discussion is the mechanism behind increases in two subtypes of BPV and impairment of BRS in rats infused with NA. As mentioned above, BP elevation by NA infusion could have been a factor in this study, because both azelnidipine and hydralazine lowered BP in NA-infused rats, alleviating augmentation of BPV.

It would be interesting to discuss the present findings in reference to BP abnormality seen in patients with pheochromocytoma, from which noradrenaline and/or adrenaline is excessively secreted into the blood stream [20–22]. This BP abnormality is often referred to as being labile or paroxysmal [21]. It was reported that increased catecholamine level modulated function of the baroreflex loop including the vasomotor center of the brain in pheochromocytoma patients [23]. In the present study, it is possible that infused NA acts on the baroreflex loop including the central vasomotor center, leading to impaired BRS and/or increased BPV. Azelnidipine was reported to inhibit outflow of the sympathetic nervous system (SNS) [24], and indeed, it lowered heart rate of the dark cycle at day 14 in NA-infused rats in the present study. It seems, however, unlikely that this inhibitory action of azelnidipine significantly contributed to alleviation of BPV in this study, because it is an inhibition of the basal activity of the SNS, but probably not related with stabilization of BP level by the baroreflex loop.

Comparing the two vasodilators used in this study, azelnidipine, a long-acting CCB, appears superior to hydralazine in alleviating NA-induced BPV and impaired BRS despite a smaller reduction in BP. As discussed above, BP reduction by both vasodilators was probably a factor associated with alleviated BPV, while we may need to consider a BP-lowering-independent effect for azelnidipine. CCBs were reported to increase elasticity of the large conduit vessels, reducing central arterial pressure and/or pulse pressure [25, 26]. Indeed, in the present study, azelnidipine significantly reduced pulse pressure levels of NA-infused rats at the light cycles despite similar or smaller BP-lowering effects, as compared with hydralazine (Fig. 1, Supplementary Table 3). Increased elasticity likely improves pulsatile stretching of vascular walls of the aortic arch and/or carotid arteries, leading to preserved function of baroreceptors. To look at morphological alterations in vascular walls, we microscopically measured medial thickness of the aortic arch, but no difference was found in the inhibitory effects on NA-induced medial thickening between azelnidipine and hydralazine (data not shown). Therefore, functional analyses, such as ultrasound measurements of elasticity and/or follow-mediated dilatation of the vessels, are necessary to clarify the mechanisms of action.

Lastly, we need to mention the weaknesses and/or limitations of the present study. First, azelnidipine and hydralazine were given via rat chow and drinking water, respectively. Because food and water consumption during the dark cycle was higher than during the light cycle, there should have been differences in doses of the drugs given between the two cycles. According to Fig. 1 and Supplementary Tables 1 and 2, the BP-lowering effects of azelnidipine during the light cycle were similar to those during the dark, a finding consistent with the notion of the long-acting feature and/or the long plasma half-life of this CCB [27]. Meanwhile, hydralazine lowered BP more efficiently during the dark cycle compared to the light, probably because of the shorter plasma half-life [28]. So, we need to be careful in discussing different efficacies of two vasodilators observed in this study. Second, we used young rats (8 weeks old), while augmented BPV is often a clinical issue for elderly patients with hypertension or cardiovascular diseases. In addition, the present rat model was made by NA infusion only for 14 days, while patients showing augmented BPV have sometimes a years-long history of hypertension. These points should be taken into account in interpreting the present findings.

### Supplementary information

Supplementary information
