# Supplementary material for: Effects of vasodilators on beat-to-beat and every fifteen minutes blood pressure variability induced by noradrenaline infusion in rats
Source: Hypertens Res. 2024 Feb 9;47(4):1017–23. doi: 10.1038/s41440-024-01595-w (PMC10994831; doi:10.1038/s41440-024-01595-w)
Supplement: Supplementary file 1 — Supplementary information [file 41440_2024_1595_MOESM1_ESM.docx]

**Supplementary Table 1.** Averages of systolic and diastolic blood pressure (SBP and DBP) during 12-h time period of the light cycles before and 7 and 14 days of vehicle or noradrenaline (NA) infusion with or without oral administration of azelnidipine (Azl) or hydralazine (Hyd).

SBP (mmHg)

|  | Before | Day 7 | Day 14 |
| --- | --- | --- | --- |
| Control | 117.8 ± 1.5 | 119.0 ± 2.3 | 118.6 ± 3.7 |
| NA | 116.9 ± 1.7 | 144.7 ± 2.9** | 146.7 ± 6.4** |
| NA + Azl | 114.3 ± 1.4 | 130.2 ± 1.8^++^ | 132.3 ± 2.3 |
| NA + Hyd | 114.7 ± 4.8 | 128.4 ± 4.8^++^ | 132.2 ± 6.3 |

DBP (mmHg)

|  | Before | Day 7 | Day 14 |
| --- | --- | --- | --- |
| Control | 80.8 ± 0.8 | 82.0 ± 2.2 | 82.1 ± 2.7 |
| NA | 79.4 ± 0.6 | 91.4 ± 2.1* | 93.2 ± 4.0 |
| NA + Azl | 77.9 ± 1.2 | 85.5 ± 2.0 | 88.5 ± 2.6 |
| NA + Hyd | 76.7 ± 3.9 | 80.3 ± 3.4^+^ | 86.9 ± 4.8 |

Mean ± SEM; **P*<0.05, ***P*<0.01, vs. control group; ^+^*P*<0.05, ^++^*P*<0.01, vs. NA group without vasodilator treatment.

**Supplementary Table 2.** Averages of systolic and diastolic blood pressure (SBP and DBP) during 12-h time period of the dark cycles before and 7 and 14 days of vehicle or noradrenaline (NA) infusion with or without oral administration of azelnidipine (Azl) or hydralazine (Hyd).

SBP (mmHg)

|  | Before | Day 7 | Day 14 |
| --- | --- | --- | --- |
| Control | 122.8 ± 2.2 | 124.0 ± 4.2 | 123.2 ± 4.3 |
| NA | 121.1 ± 1.8 | 145.3 ± 4.2** | 146.5 ± 5.6** |
| NA + Azl | 118.6 ± 1.4 | 132.8 ± 1.6 | 135.0 ± 2.0 |
| NA + Hyd | 117.6 ± 6.0 | 122.5 ± 5.5^++^ | 126.9 ± 5.8^++^ |

DBP (mmHg)

|  | Before | Day 7 | Day 14 |
| --- | --- | --- | --- |
| Control | 86.9 ± 1.1 | 88.0 ± 3.5 | 87.8 ± 3.1 |
| NA | 84.0 ± 0.7 | 94.9 ± 3.5 | 95.8 ± 3.7 |
| NA + Azl | 82.8 ± 1.0 | 88.7 ± 2.3 | 91.1 ± 2.7 |
| NA + Hyd | 81.1 ± 4.8 | 77.7 ± 3.3^++^ | 83.4 ± 3.7 |

Mean ± SEM; ***P*<0.01, vs. control group; ^++^*P*<0.01, vs. NA group without vasodilator treatment.

**Supplementary Table 3.** Averages of pulse pressure during 12-h time period of the light and dark cycles before and 7 and 14 days of vehicle or noradrenaline (NA) infusion with or without oral administration of azelnidipine (Azl) or hydralazine (Hyd).

Pulse Pressure of the light cycles (mmHg)

|  | Before | Day 7 | Day 14 |
| --- | --- | --- | --- |
| Control | 37.0 ± 1.5 | 37.1 ± 0.9 | 36.5 ± 2.2 |
| NA | 37.5 ± 1.3 | 53.4 ± 1.9** | 53.5 ± 3.1** |
| NA + Azl | 36.4 ± 0.7 | 44.7 ± 1.0^++^ | 43.7 ± 1.1^+^ |
| NA + Hyd | 38.0 ± 1.4 | 48.2 ± 1.7 | 45.2 ± 3.2 |

Pulse Pressure of the dark cycles (mmHg)

|  | Before | Day 7 | Day 14 |
| --- | --- | --- | --- |
| Control | 35.9 ± 1.5 | 36.0 ± 1.2 | 35.4 ± 2.3 |
| NA | 37.1 ± 1.6 | 50.4 ± 2.2** | 50.7 ± 3.0** |
| NA + Azl | 35.8 ± 0.9 | 44.2 ± 1.3 | 43.9 ± 1.6 |
| NA + Hyd | 36.5 ± 1.7 | 44.9 ± 2.4 | 43.5 ± 3.5 |

Mean ± SEM; ***P*<0.01, vs. control group; ^+^*P*<0.05, ^++^*P*<0.01, vs. NA group without vasodilator treatment.
